# Dataset on the absorption of PCDTBT:PC_70_BM layers and the electro-optical characteristics of air-stable, large-area PCDTBT:PC_70_BM-based polymer solar cell modules, deposited with a custom built slot-die coater

**DOI:** 10.1016/j.dib.2017.01.003

**Published:** 2017-01-11

**Authors:** Dimitar I. Kutsarov, Edward New, Francesco Bausi, Alina Zoladek-Lemanczyk, Fernando A. Castro, S. Ravi P. Silva

**Affiliations:** a NanoElectronics Centre, Advanced Technology Institute, Department of Electrical & Electronic Engineering, University of Surrey, Guildford, Surrey GU2 7XH, UK; b National Physical Laboratory (NPL), Teddington, Middlesex, TW11 0LW, UK

**Keywords:** PCDTBT:PCBM, Slot-die coating, Organic Solar Cell, Large-area

## Abstract

The data presented in this article is related to the research article entitled “Fabrication of air-stable, large-area, PCDTBT:PC_70_BM polymer solar cell modules using a custom built slot-die coater” (D.I. Kutsarov, E. New, F. Bausi, A. Zoladek-Lemanczyk, F.A. Castro, S.R.P. Silva, 2016) [1]. The repository name and reference number for the raw data from the abovementioned publication can be found under: https://doi.org/10.15126/surreydata.00813106. In this data in brief article, additional information about the absorption properties of PCDTBT:PC_70_BM layers deposited from a 12.5 mg/ml and 15 mg/ml photoactive layer dispersion are shown. Additionally, the best and average J-V curves of single cells, fabricated from the 10 and 15 mg/ml dispersions, are presented.

**Specifications Table**

**Table:** 

Subject area	Electronic Engineering, Physics
More specific subject area	Nanotechnology, Renewable energy, Material science
Type of data	Figures
How data was acquired	Optical characterization and Electro-optical measurement of device characteristics
Data format	Plotted and analyzed
Experimental factors	
Experimental features	The layers with the photoactive ink were deposited on glass substrates with a custom built slot-die coater and their absorption spectra was measured. Complete solar cells were fabricated and characterized with a solar simulator at 1 Sun. The data plotted represents the raw measurement data.
Data source location	No GPS signal and therefore coordinates could be obtained in the Clean room of the ATI at the University of Surrey. Nevertheless, according to Google maps, the coordinates are 51 °14׳36.0"N 0 °35׳39.2"W
Data accessibility	The data is with this article. A detailed excel spreadsheet with the raw data for the original article by Kutsarov et al. [1], however, can be found in the public repository here: https://doi.org/10.15126/surreydata.00813106

**Value of the data**

• A custom built slot-die coater was used to deposit PCDTBT:PCBM layers from dispersions with different concentrations in order to optimize coating parameters and accomplish the deposition of reproducible and homogeneous layers at different temperatures.
• PCDTBT:PCBM-based single cells were fabricated from different photoactive layer inks to investigate the effect of the dispersion concentration on the device characteristics.
• The data can be used as a comparison and a benchmark for other researchers, who work on the field of fabrication of large-area polymer solar cells.

## 1 Data

### 1.1 Thin film characterization

**Fig. 1:**
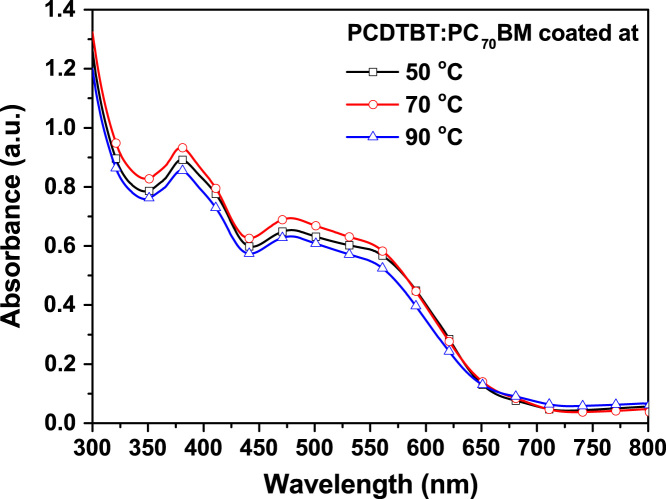
Average UV–vis spectra of PCDTBT:PC_70_BM layers coated at different temperatures for a fixed solution concentration of 15 mg/mL. The UV–vis spectra were measured at position P2 for each sample as reported by Kutsarov et al. [1].

**Fig. 2:**
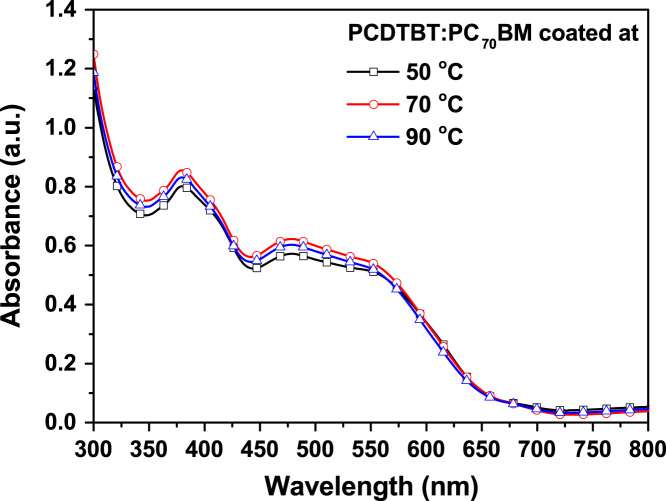
Average UV–vis spectra of PCDTBT:PC_70_BM layers coated at different temperatures for a fixed solution concentration of 12.5 mg/mL. The UV–vis spectra were measured at position P2 for each sample as reported by Kutsarov et al. [1].

The dataset in this article shows a dependence of the PCDTBT:PCBM layer properties on the deposition temperature. This is directly related to an alteration of the optical density of the deposited film and hence, the thickness of the films in Figs. 1 and 2. The measured thickness for photoactive inks with concentrations of 10, 12.5, and 15 mg/ml, which were deposited at 50, 70, and 90 °C, are reported by Kutsarov et al. [1], whereas the raw data, used for the compiling of the graph, is furthermore included in the excel spread sheet in the public repository.

## 2 Solar cell characterization

**Fig. 3:**
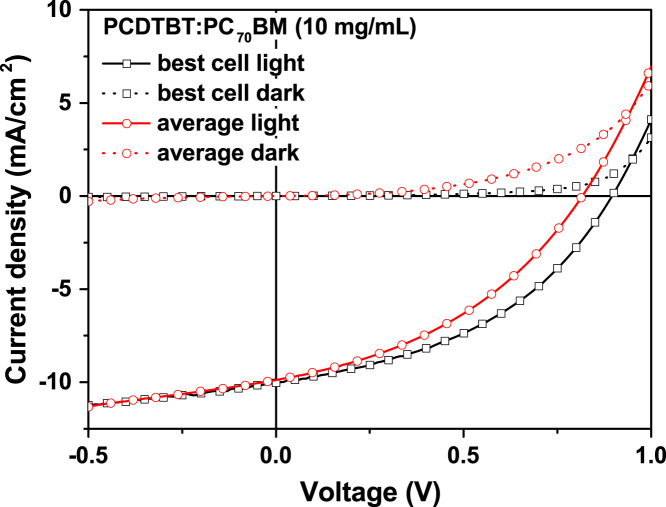
J–V characteristics of the best performing single cell (based on 12 cells from 2 modules) and the average J–V curve of 12 single cells under illumination and in the dark as reported by Kutsarov et al. [1].

**Fig. 4:**
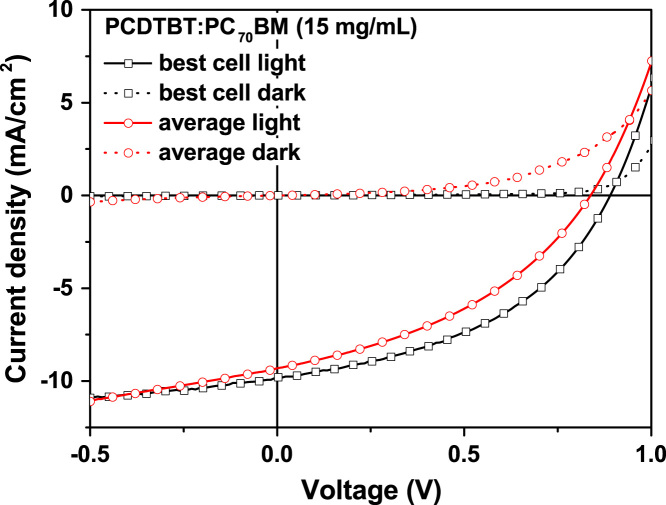
J–V characteristics of the best performing single cell (based on 6 cells from 1 module) and the average J–V curve of 6 single cells under illumination and in the dark as reported by Kutsarov et al. [1].

The J–V characteristics of PCDTBT:PC_70_BM based devices, which were fabricated from a 10 and 15 mg/ml photoactive layer dispersion, are shown in Figs. 3 and 4, respectively. The detailed device characteristics are shown in Supplementary information Table S1 and S2 by Kutsarov et al. [1]. It can be seen that the average characteristics of the cells are reduced, compared to the characteristics of the best performing cell. This is due to fabrication defects, as reported by Kutsarov et al. [1].

## 3 Experimental design, materials and methods

### 3.1 Materials

For the optical characterization in this data in brief, PCDTBT:PC_70_BM layers were deposited with a custom built slot-die coater on glass substrates. The photoactive layer ink consisted of the donor material PCDTBT (SOL 4280, Solaris Chem Inc.) and [6]-Phenyl-C71-butyric acid methyl ester (PC_70_BM, Solenne BV) as the acceptor material. PCDTBT and PC_70_BM were mixed in a 1:4 wt ratio and dispersed in a 3:1 solvent mixture by volume of anhydrous o-DCB and CB (1,2-dichlorobenzene and chlorobenzene) to achieve a total concentration of 35 mg/mL [1]. The donor-acceptor mixture was stirred at room temperature until complete dissolution and then diluted to a desired concentration (10, 12.5, or 15 mg/ml).

## 4 Sample and solar cell fabrication

Prior to the deposition of the PCDTBT:PC_70_BM dispersion, the glass substrates were cleaned in an ultrasonic bath sequentially for 5 min in Decon 90 detergent solution with deionized water (DI), DI, acetone, and methanol. Then, the substrates were blown dry with a nitrogen gun and treated with an oxygen plasma for 5 min (100 W, 15 sccm O2, Emitech K1050X plasma cleaner). The PCDTBT:PC_70_BM layers were deposited under ambient conditions on top of the glass substrate, at a substrate temperature of 50 °C, 70 °C, or 90 °C. A flow rate of 100 µl/min, coating speed of 18 mm/s, and a screw gap of 200 µm were used. After the deposition, the layers were annealed for 10 min at 70 °C to ensure the evaporation of any excess solvents. The complete devices comprised an inverted structure of glass-ITO/ZnO/PCDTBT:PC_70_BM/MoO3/Al and were fabricated according to the experimental details reported by Kutsarov et al. [1].

## 5 Methods

To characterize the PCDTBT:PC_70_BM layers absorption spectra, UV–visible (UV–vis) spectroscopy was used (Varian Cary 5000 UV–vis–NIR spectrophotometer). The spectra were recorded through a shadow mask with a circular opening (diameter of 5 mm), which was placed at the center of the slot-die coated layer as reported by Kutsarov et al. [1]. The reported spectra represent the average of two spectra, measured for each sample in the wavelength range from 300 nm to 900 nm relative to a glass reference.

Current–voltage (I–V) characterization was conducted using a Keithley 2400 source measurement apparatus in a four-wire setup in ambient atmosphere with an ORIEL solar simulator (class ABA) at AM 1.5 G. A silicon reference cell (PV Measurements, Inc. 20 mm×20 mm) was used for the calibration of the illumination source to 1 Sun (100 mW/cm^2^).
